# Cortico-hippocampal networks underpin verbal memory encoding in temporal lobe epilepsy

**DOI:** 10.1093/braincomms/fcaf067

**Published:** 2025-02-12

**Authors:** Giorgio Fiore, Davide Giampiccolo, Fenglai Xiao, Matthias J Koepp, Juan E Iglesias, Sjoerd B Vos, Jane de Tisi, Andrew W McEvoy, Giulio A Bertani, Marco Locatelli, Roisin Finn, Lorenzo Caciagli, Meneka Sidhu, Marian Galovic, Sallie Baxendale, John S Duncan, Anna Miserocchi

**Affiliations:** Victor Horsley Department of Neurosurgery, National Hospital for Neurology and Neurosurgery, London WC1N 3BG, UK; Unit of Neurosurgery, IRCCS Ca’ Granda Foundation Ospedale Maggiore Policlinico, Milan 20122, Italy; Victor Horsley Department of Neurosurgery, National Hospital for Neurology and Neurosurgery, London WC1N 3BG, UK; Department of Clinical and Experimental Epilepsy, UCL Queen Square Institute of Neurology, University College London, London WC1N 3BG, UK; Department of Clinical and Experimental Epilepsy, UCL Queen Square Institute of Neurology, University College London, London WC1N 3BG, UK; Department of Clinical and Experimental Epilepsy, UCL Queen Square Institute of Neurology, University College London, London WC1N 3BG, UK; Athinoula A. Martinos Center for Biomedical Imaging, Massachusetts General Hospital and Harvard Medical School, Boston, MA 02129, USA; Department of Computer Science, Center for Medical Image Computing, University College London, London WC1E 6BT, UK; Department of Computer Science, Center for Medical Image Computing, University College London, London WC1E 6BT, UK; Neuroradiological Academic Unit, UCL Queen Square Institute of Neurology, University College London, London WC1N 3BG, UK; Centre for Microscopy, Characterisation, and Analysis, The University of Western Australia, Nedlands 6009, Australia; Victor Horsley Department of Neurosurgery, National Hospital for Neurology and Neurosurgery, London WC1N 3BG, UK; Victor Horsley Department of Neurosurgery, National Hospital for Neurology and Neurosurgery, London WC1N 3BG, UK; Unit of Neurosurgery, IRCCS Ca’ Granda Foundation Ospedale Maggiore Policlinico, Milan 20122, Italy; Unit of Neurosurgery, IRCCS Ca’ Granda Foundation Ospedale Maggiore Policlinico, Milan 20122, Italy; Victor Horsley Department of Neurosurgery, National Hospital for Neurology and Neurosurgery, London WC1N 3BG, UK; Department of Clinical and Experimental Epilepsy, UCL Queen Square Institute of Neurology, University College London, London WC1N 3BG, UK; Department of Bioengineering, University of Pennsylvania, Philadelphia, PA 19104, USA; Department of Clinical and Experimental Epilepsy, UCL Queen Square Institute of Neurology, University College London, London WC1N 3BG, UK; MRI Unit, Epilepsy Society, Chalfont Centre for Epilepsy, Chalfont St Peter, Gerrards Cross SL9 ORJ, UK; Department of Clinical and Experimental Epilepsy, UCL Queen Square Institute of Neurology, University College London, London WC1N 3BG, UK; Department of Neurology, Clinical Neuroscience Center, University Hospital and University of Zurich, Zurich 8057, Switzerland; Department of Clinical and Experimental Epilepsy, UCL Queen Square Institute of Neurology, University College London, London WC1N 3BG, UK; Department of Clinical and Experimental Epilepsy, UCL Queen Square Institute of Neurology, University College London, London WC1N 3BG, UK; MRI Unit, Epilepsy Society, Chalfont Centre for Epilepsy, Chalfont St Peter, Gerrards Cross SL9 ORJ, UK; Victor Horsley Department of Neurosurgery, National Hospital for Neurology and Neurosurgery, London WC1N 3BG, UK

**Keywords:** seizure, learning, cortex, hippocampus, theory

## Abstract

Knowledge of the structural underpinnings of human verbal memory is scarce. Understanding the human verbal memory network at a finer anatomical scale will have important clinical implications for the management of patients with verbal memory impairment. In this cross-sectional study, we aimed to assess the contributions of cerebral cortex and hippocampal subfields to verbal memory encoding in temporal lobe epilepsy. We included consecutive patients (*n* = 84) with radiologically and pathologically defined hippocampal sclerosis (HS) (44 left-sided) and unilateral temporal lobe epilepsy, and healthy volunteers (*n* = 43) who were comparable regarding age and sex. The morphometric and volumetric measures of cerebral cortex and hippocampal subfields were extracted from high-resolution MRI scans. People included in this study underwent standardized neuropsychological evaluation, including measures of verbal memory assessed through the Adult Memory and Information Processing Battery. Verbal memory performances were *Z*-scores corrected by using means and standard deviations published for sample standardization. Associations between verbal learning *Z*-scores and the grey matter volume of the cerebral cortex and hippocampal subfields were investigated. Reduction of grey matter volumes in the left and right medial and dorsolateral prefrontal cortex (*P*_corr_ < 0.0001), superior and middle temporal gyri (*P*_corr_ < 0.0001), anterior and posterior cingulate cortex (*P*_corr_ < 0.0001) and of the left ventrolateral prefrontal cortex (*P*_corr_ < 0.0001) and parietal–temporal–occipital junction (*P*_corr_ < 0.0001) were associated with worse verbal learning. These findings were consistent across both the entire cohort and in a subgroup analysis focused exclusively on HS patients. Within hippocampi, smaller volumes of the left dentate gyrus (*P* = 0.003), cornu ammonis 4 (*P* = 0.005) and cornu ammonis 3 (*P* = 0.03) were associated with worse verbal learning *Z*-scores. This study demonstrates that verbal learning in patients with temporal lobe epilepsy is strongly related to the volume of distinct regions of the prefrontal, temporal and cingulate cortices and left dentate gyrus, cornu ammonis 4 and cornu ammonis 3 hippocampal subfields. It provides the basis to suggest a corticohippocampal network for verbal learning in these patients, improving our understanding of human verbal memory. These biomarkers may inform attractive targets for forthcoming modulating therapies. Future work may also analyse the impact of sparing part of the left dentate gyrus, cornu ammonis 4 and cornu ammonis 3 as a protective measure against verbal memory impairment after surgery for temporal lobe epilepsy.

## Teaser

In this cross-sectional study of 84 hippocampal sclerosis patients and 43 healthy controls, Fiore *et al*. linked verbal learning variability to cortical and hippocampal morphometry. Smaller volumes in prefrontal, temporal, cingulate cortices and left dentate gyrus, CA4 and CA3 were associated with poorer verbal learning in temporal lobe epilepsy.

## Graphical Abstract

**Graphical Abstract:**
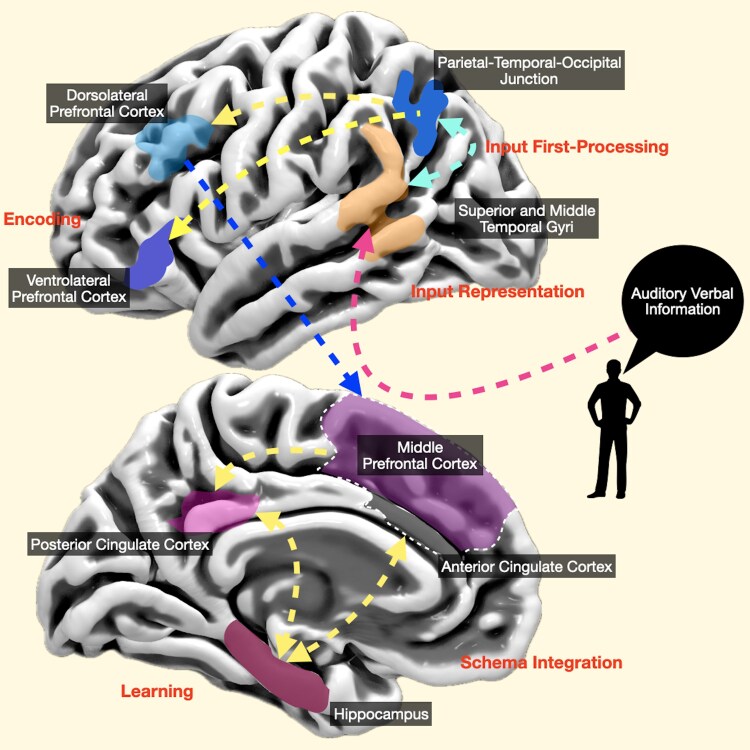

## Introduction

The structural basis of human verbal memory is poorly understood.

It is accepted that the medial temporal lobe (MTL) is critically involved in human memory, relying on its ability to store and retrieve information.^1^ Impairment of episodic memory encoding is common in individuals with drug-resistant temporal lobe epilepsy (TLE),^2^ and anterior temporal lobe resection (ATLR) is associated with postoperative verbal memory decline in up to 60% of patients.^3,4^ However, the considerable individual variation in the extent and direction of verbal memory changes after ATLR suggests that there may be variation in the structural and functional networks supporting verbal memory.^5^

Processing verbal material engages multiple areas of the cerebral cortex to perceive, maintain and store information.^6^ The temporal cortex is hypothesized to serve a crucial role as an initial processing unit.^7^ Given the association between verbal learning and the acoustic properties of words,^8-10^ the temporal cortex may be essential for integrating acoustic and phonemic features during the initial stages of verbal memory encoding.^10^ Functional investigations indicate that the posterior temporal cortex, parietal–temporal–occipital junction (PTOj) and ventrolateral prefrontal cortex (vlPFC) are integral to the processing of verbal material during learning tasks,^10-12^ with frontal lobe contributions to memory encoding having recently become better understood.^13^ Neurophysiological and lesion studies have shown that increased activity of the prefrontal cortices is associated with better verbal memory encoding,^6,14,15^ and patients with lesions in these regions have verbal learning impairment.^16^ The cortical regions studied, however, were limited.

Also, the role of hippocampal subfields in verbal memory encoding needs clarification. Functional MRI (fMRI) studies showed that increased functional activation in the left hippocampus relates to better verbal memory preoperative scores,^17^ with lateralization of verbal memory activation being found to be the best predictor of verbal learning change after ATLR.^18^ The low spatial resolution of fMRI, however, does not allow the determination of the contribution of the various hippocampal subfields to verbal memory. Moreover, fMRI analyses can be affected by the coactivation of multiple brain regions, especially in patients with lesions.^17,19^ Pathological studies offer the potential to investigate the impact of the damage of hippocampal subfields on patients’ verbal memory performances.^20-23^ However, a quantitative relationship between the extent of hippocampal subfield degeneration and verbal memory performances is hard to establish with histology. The most common pattern of hippocampal cell loss, ILAE HS Type 1, manifests as predominant loss of neurons and gliosis in CA1 and CA4 subfields.^24,25^ Patterns of hippocampal sclerosis (HS) appear to be related to various clinical aspects of TLE and may have significance for postoperative prognosis of seizure outcome, but not cognitive outcomes.^26,27^

As above, both the cerebral cortex and hippocampus are established contributors to verbal learning. Animal studies suggest the cingulate cortex may serve as a direct pathway linking the hippocampus and these cortical regions.^28,29^ However, the extent to which the cingulate regions contribute to verbal learning remains to be fully elucidated.

Using voxel-based morphometry (VBM)^30^ and volumetric analyses of the hippocampus and its subfields,^31-33^ we aimed to investigate the anatomical basis of verbal memory encoding function and dysfunction in the cerebral cortex and the hippocampus of patients with TLE, in an objective and quantitative way. We hypothesized that grey matter atrophy of cortical regions involved in the perception of verbal material (e.g. temporal cortices) and in memory encoding (e.g. prefrontal cortices), as well as in regions that connect the neocortex and allocortex (e.g. the cingulum), would be associated with poorer verbal learning performance. Since there is evidence of predominantly left hippocampal lateralization for verbal memory,^17,18,34^ and based on human episodic memory models postulating the involvement of dentate gyrus (DG),^7,35,36^ CA4^37,38^ and CA3,^35,36,39^ for learning and memory encoding, we also hypothesized that a reduction of the volumes of the subfields in the left hippocampus will correlate with impairment in verbal learning.

## Materials and methods

### Study design

This is a cross-sectional study involving morphometric and volumetric measures of cerebral cortex and hippocampal grey matter structures, in relation to human verbal memory encoding. We studied 84 HS patients (44 left-sided) included in our surgical epilepsy programme between January 2007 and November 2014 and 43 healthy control (HC) subjects. The clinical data, fully anonymized, were extracted from prospectively maintained databases at the National Hospital for Neurology and Neurosurgery (NHNN), London. The data included patients’ clinical history, examination, MRI findings, side of epileptogenic zone, number of antiseizure medications (ASMs), pathology^2^ and presurgical neuropsychological scores.^40^ Inclusion criteria for the current investigation were radiologically and pathologically defined evidence of HS, available preoperative scores on verbal memory tests and preoperative high-resolution structural MRI scans. HCs were volunteers with no history of neurological or psychiatric disease. This study was approved by the Health Research Authority Ethics Committee (22/SC/0016) and followed the STROBE guidelines for cross-sectional studies.^41^

### Neuropsychological measures

Patients and controls received extensive standardized neuropsychological evaluation, including measures of verbal memory. Since verbal learning has clinical validity in patients with HS,^5^ the list learning task scores from the Adult Memory and Information Processing Battery (AMIPB) were used as measures of verbal memory in the study population. In the verbal learning task, subjects are read a list of 15 words and asked to recall as many as possible. After five trials, the total number of recalled words is recorded, and the percentage represents the score. The raw scores from the verbal learning test were converted to *Z*-scores using the means and standard deviations published for sample standardization.

### MRI acquisition

The participants included in this study underwent structural high-resolution preoperative MRI on the same scanner (3T General Electric Excite HDx) at the Epilepsy Society, Chalfont, UK. The 3D T1 fast spoiled gradient echo scan was acquired with the following parameters: repetition time = 6.6 ms, echo time = 2.8 ms, inversion time = 450 ms, matrix = 256 × 256 × 192, for a voxel size of 0.9375 × 0.9375 × 1.1 mm.

### Morphometric analysis

Voxel-based morphometry analyses were performed using the Computational Anatomy Toolbox (CAT) 12 (https://neuro-jena.github.io/cat/) run within Statistical Parametric Mapping (SPM) 12 (Wellcome Centre for Human Neuroimaging, London, UK; https://www.fil.ion.ucl.ac.uk/spm/software/spm12/). Briefly, CAT12 preprocessed and tissue-segmented individuals’ structural images. Then, spatial registration to the MNI 152 template was obtained through Geodesic Shooting. Grey matter differences were assessed through a multiple linear regression model. Verbal memory *Z*-scores were included as the main explanatory variable. Total intracranial volume (TIV), age, sex, frequency of focal unaware seizures, history of epilepsy, duration of epilepsy and the number of ASMs were used as covariates. A Bonferroni corrected statistical threshold of *P* < 0.05 was employed. To control for false positive findings and ensure the identification of meaningful cortical regions,^42^ a minimum cluster size of 100 contiguous voxels was applied. All data were quality controlled according to procedures implemented in CAT12 to address scans’ misalignment, misregistration or inaccurate thickness estimation. No anatomical masks were employed.

After analysing the entire cohort of patients with HS and HCs, we conducted a subgroup analysis focusing exclusively on HS patients. To assess the spatial overlap between the regions identified in the full cohort (HS patients and HCs) and the HS-only subgroup, we conducted a voxel-wise conjunction analysis in SPM. First, we obtained thresholded statistical parametric maps (*P* < 0.05, corrected) from each model. We then produced a binary mask representing significant clusters for each analysis and multiplied these masks to generate a conjunction map. This procedure isolates voxels that survive statistical thresholding in both models simultaneously, ensuring that any reported overlap reflects regions consistently associated with verbal memory, regardless of the inclusion of HCs.

### Volumetric analysis

**Figure 1:**
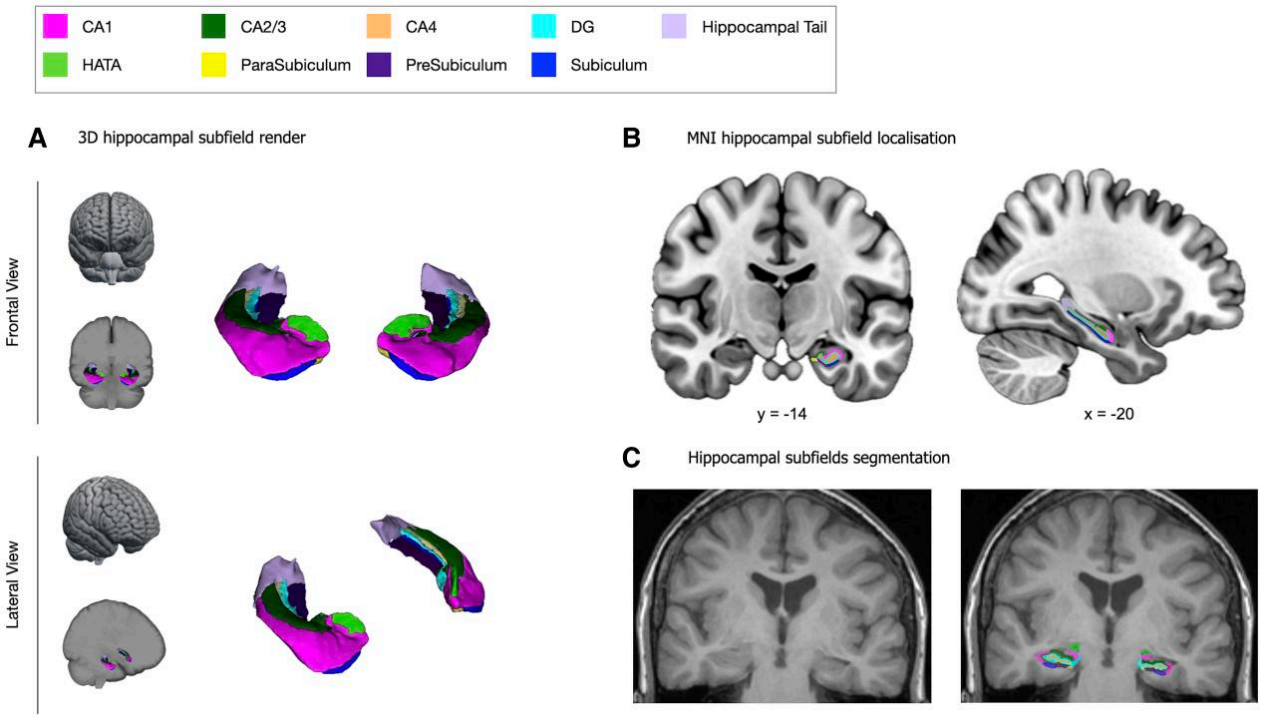
**Hippocampal subfields.** Localization of hippocampal subfields shown in a 3D brain representation (**A**) and the MNI 152 template (**B**). The automated segmentation of the hippocampal subfields displayed on a structural T1 MRI coronal slice (**C**). Both the 3D reconstruction and T1 MRI segmentation of hippocampal subfields are from a patient with HS and verbal memory deficit (AMIPB *Z*-score: −2 SD). CA, cornu ammonis; DG, dentate gyrus; HATA, hippocampus–amygdala-transition-area; MNI, Montreal Neurological Institute.

The segmentation of the whole hippocampus was obtained through Hipposeg software (http://niftyweb.cs.ucl.ac.uk/program.php?p=HIPPOSEG) which exploits a multiatlas-based segmentation propagation method using STAPLE (simultaneous truth and performance level estimation). Hipposeg is established for the segmentation variable morphology, including sclerotic hippocampi.^33^ As previously shown, Hipposeg delineates the hippocampus with no more variability than expert human raters. The segmentation masks were reviewed by two independently trained raters (G.F. and M.G.) and misclassifications were corrected as previously described.^43,44^ The estimated hippocampal volumes were corrected for TIV according to a well-established formula.^33^ TIV was extracted using a parcellation algorithm based on Geodesic Information Flows.^45^ Then, the segmentations of the hippocampal subfields were obtained using FreeSurfer by J.E.I. and S.B.V.^32^ The volumes of the following hippocampal grey matter structures were extracted, TIV-corrected, double-checked and included in the volumetric analysis: CA1, CA3, CA4, DG, subicular complex (para-, pre- and subiculum), hippocampus–amygdala-transition-area (HATA) and hippocampal tail (Fig. 1).

### Statistical analysis

Frequencies were reported as a percentage and compared by χ^2^ and Fisher exact tests according to sample size. Continuous normally distributed variables were reported as mean ± standard deviation (SD) and compared with Student’s *t*-test or analysis of variance. Continuous nonparametrically distributed variables were reported as the median and interquartile range (IQR) and compared via the Mann–Whitney U-test and Kruskal–Wallis test. Linear regression models were used to assess the predictive value of hippocampus and hippocampal subfields’ volumes on verbal learning *Z*-scores. Multiple linear regression models were employed to correct for covariates. The covariates TIV, age, sex, handedness, history, duration of epilepsy, frequency of seizures, presence and side of HS and number of ASM were chosen based on the literature. Multicollinearity was assessed at each step through tolerance and the variance inflation factor (VIF). Residuals were checked for normality. Statistical significance was regarded for *P*-values <0.05. Bootstrapping with 1000 samples was employed to control and check the stability of the multivariate models. In the case of more hippocampal subfields predicting verbal memory scores, we planned to use backward stepwise regression methods to identify the regression model and subfield(s) with the highest predictive value. Receiver operating characteristic (ROC) analyses were used to inspect the diagnostic value of the significant subfields in identifying patients with HS and verbal memory impairment. All statistical analyses were performed using IBM SPSS (version 28.0, International Business Machines Corp, New York, USA) and R software 4.2.2 (R Foundation for Statistical Computing, Vienna, Austria; http://www.r-project.org/index.html).

## Results

The study included 127 subjects. The clinical and demographical features of the participants are summarized in Table 1. The control population was well matched for age (*P* = 0.67) and sex (*P* = 0.85) to the HS population.

**Table 1:** Study population features

Variable	Left HS (*N* = 44)	Right HS (*N* = 40)	HC (*N* = 43)	*P*-value
Age (years)	37.7 ± 11.9	38.2 ± 11	36.1 ± 11.8	0.67
Duration (years)	24.6 ± 14	27.2 ± 13.7		0.4
Sex (female)	26 (59%)	26 (65%)	26 (60.5%)	0.85
Verbal learning *Z*-score	−1.22 ± 0.9	−0.8 ± 0.9	0.4 ± 0.8	<0.001*
Handedness (left/right)	11/33	4/36	7/36	0.19

^*^ *P* < 0.05.

### Voxel-based morphometry

**Figure 2:**
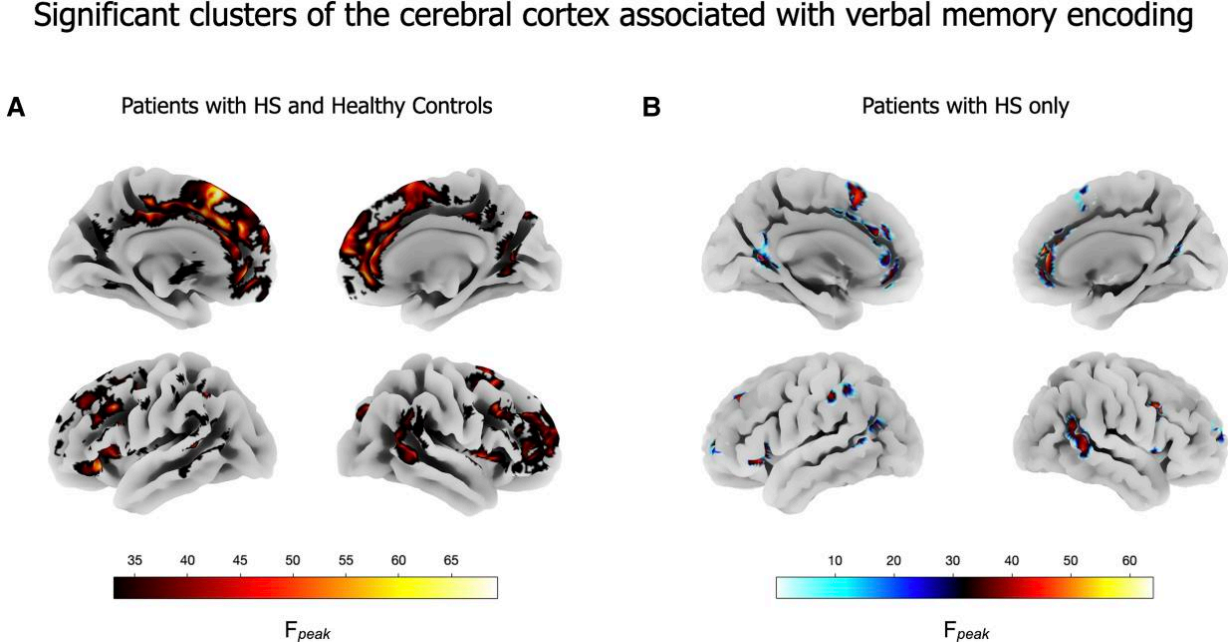
**Significant clusters of the cerebral cortex associated with verbal memory encoding.** Cortical representation of brain cortex clusters where greater grey matter volume was associated with better verbal memory performance in the entire (*N* = 127) cohort (**A**), including patients with HS and HCs, as well as in the subgroup analysis of HS patients (*N* = 84) alone (**B**). Multiple linear regression models were used, and an *F*-score thresholded map of the significant clusters (*P* < 0.05, Bonferroni corrected) is shown. The regression model that included patients with HS and HCs was constructed as follows: grey matter volume = *β*_0_ + *β*_1_⋅(verbal memory *Z*-score) + *β*_2_⋅(age) + *β*_3_⋅(sex) + *β*_4_⋅(frequency of focal seizures by year) + *β*_5_⋅(duration of epilepsy in years)+ *β*_6_⋅(number of ASMs) + *β*_7_⋅(TIV) + *β*_8_⋅(patient or HC group) + *ɛ*, where *β*_0_ is the intercept, *β*_1_ is the variable of interest’s coefficient, *β_n_* represents the coefficients for the covariates and *ɛ* is the error. For the model including only HS patients, the same formula was used, but without the categorical variable for group (patient or HC), since all participants in that model were HS patients.

Grey matter volume reduction of the left and right medial prefrontal cortex (mPFC) and anterior cingulate cortex (ACC) (cluster size = 30 676, *P*_corr_ < 0.0001, *F*_peak_ = 71), right dorsolateral prefrontal cortex (dlPFC; cluster size = 1675, *P*_corr_ < 0.0001, *F*_peak_ = 56), superior and middle temporal gyri (STG and MTG; right: cluster size = 2040, *P*_corr_ <0.0001, *F*_peak_ = 49; left: cluster size = 845, *P*_corr_ < 0.0001, *F*_peak_ = 45), posterior cingulate cortices (PCC; cluster size = 1217, *P*_corr_ < 0.0001, *F*_peak_ = 48) and the left dlPFC and ventrolateral PFC (vlPFC; cluster size = 30 676, *P*_corr_ < 0.0001, *F*_peak_ = 67) and PTOj (cluster size = 606, *P*_corr_ < 0.0001, *F*_peak_ = 58) were associated with worse verbal learning performances in patients with HS and HCs (Fig. 2A).

In the subgroup analysis focusing exclusively on HS patients (Fig. 2B), we found that the grey matter volume reduction of the following cortical regions was significantly associated with worse verbal learning performance: grey matter volume of the left and right mPFC and ACC (cluster size = 1681, *P*_corr_ < 0.0001, *F*_peak_ = 65), dlPFC (left: cluster size = 273, *P*_corr_ < 0.0001, *F*_peak_ = 45; right: cluster size = 388, *P*_corr_ < 0.0001, *F*_peak_ = 53), STG and MTG (right: cluster size = 900, *P*_corr_ < 0.0001, *F*_peak_ = 59; left: cluster size = 118, *P*_corr_ < 0.0001, *F*_peak_ = 41), PCC (cluster size = 503, *P*_corr_ < 0.0001, *F*_peak_ = 46) and the left vlPFC (cluster size = 1223, *P*_corr_ < 0.0001, *F*_peak_ = 53) and PTOj (cluster size = 209, *P*_corr_ < 0.0001, *F*_peak_ = 45).

**Figure 3:**
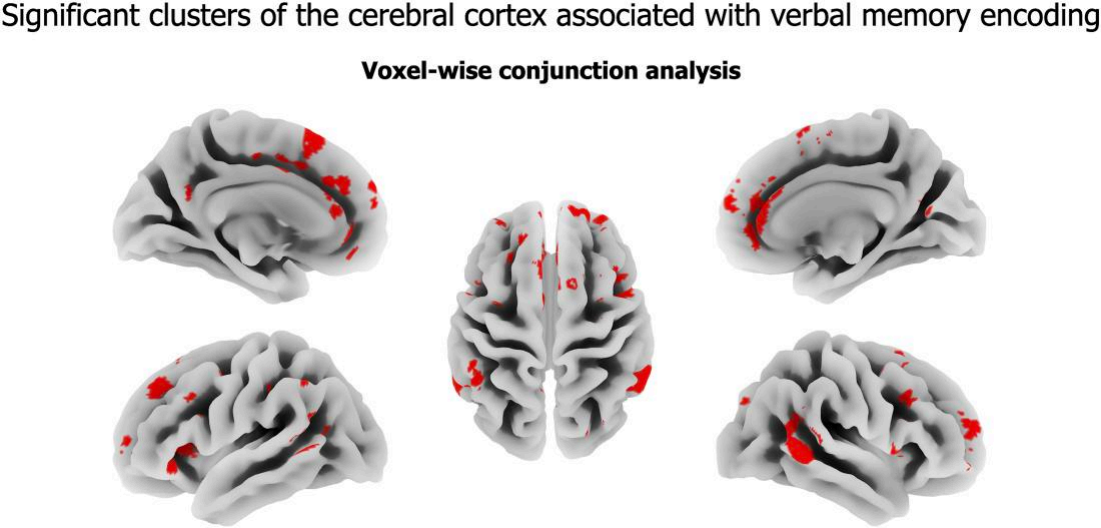
**Voxel-wise conjunction analysis.** Conjunction map illustrating the overlap of cortical regions significantly associated with verbal memory performance in both VBM analyses of the full cohort (*N* = 127; HS patients and HCs) and the subgroup restricted to HS-only patients (*N* = 84). Multiple linear regression models were run as described in Fig. 2. The resulting statistical parametric maps were thresholded at *P* < 0.05 (Bonferroni-corrected), with a minimum cluster size of 100 contiguous voxels applied. Clusters surviving statistical thresholding in both analyses are displayed.

The conjunction analysis revealed a substantial degree of spatial concordance in the significant clusters identified in the entire cohort and the HS-only subgroup, with reduced cluster sizes in the latter (Fig. 3).

### Volumetric study

The volumetric measures are available in Table 2. After correction for TIV, handedness, age, sex, history, duration of epilepsy, frequency of seizures, presence and side of HS and number of ASM, the right hippocampal volume was not associated with verbal learning *Z*-scores. In the whole cohort, the left hippocampal volume was correlated with verbal learning performance (multivariate model *R*^2^ = 0.42; *b* = 0.47; SE = 0.2; *P* = 0.03).

**Table 2:** Hippocampus and hippocampal subfields’ volumes

Subfield	Left HS	Right HS	HC	*P*-value
Left Hippocampus	1.93 ± 0.36	2.75 ± 0.32	2.77 ± 0.24	<0.001
Left DG	0.21 ± 0.04	0.31 ± 0.04	0.3 ± 0.03	<0.001
Left CA4	0.18 ± 0.04	0.26 ± 0.04	0.26 ± 0.03	<0.001
Left CA2/3	0.16 ± 0.04	0.23 ± 0.04	0.23 ± 0.03	<0.001
Left CA1	0.46 ± 0.09	0.65 ± 0.09	0.65 ± 0.07	<0.001
Left subicular C	0.56 ± 0.1	0.78 ± 0.1	0.8 ± 0.07	<0.001
Left tail	0.32 ± 0.08	0.45 ± 0.08	0.47 ± 0.08	<0.001
Left HATA	0.05 ± 0.01	0.07 ± 0.01	0.07 ± 0.01	<0.001
Right hippocampus	2.92 ± 0.3	1.93 ± 0.4	2.76 ± 0.27	<0.001
Right DG	0.33 ± 0.04	0.21 ± 0.05	0.31 ± 0.04	<0.001
Right CA4	0.28 ± 0.03	0.18 ± 0.04	0.26 ± 0.03	<0.001
Right CA2/3	0.25 ± 0.03	0.17 ± 0.03	0.24 ± 0.03	<0.001
Right CA1	0.69 ± 0.08	0.46 ± 0.1	0.64 ± 0.08	<0.001
Right subicular C	0.81 ± 0.1	0.51 ± 0.1	0.77 ± 0.08	<0.001
Right tail	0.49 ± 0.08	0.32 ± 0.08	0.49 ± 0.07	<0.001
Right HATA	0.07 ± 0.01	0.05 ± 0.01	0.07 ± 0.01	<0.001

Data are shown in cubic centimeter (cc).

**Figure 4:**
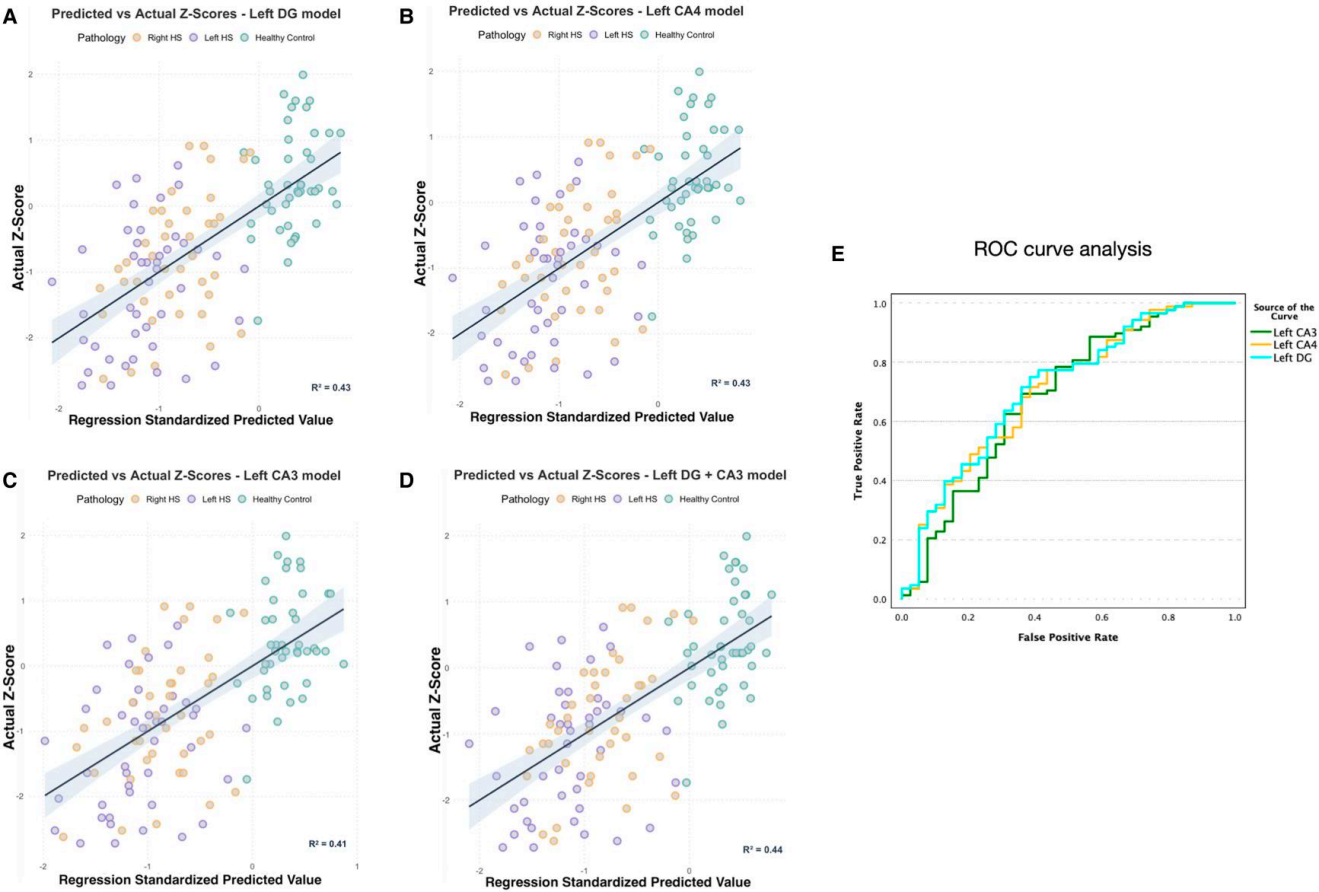
**Verbal learning *Z*-scores and hippocampal subfield volumes.** Prediction of verbal learning performances (*Z*-scores) using multiregression models including hippocampal subfield volumes in the entire cohort (*N* = 127). Each dot shows the relationship between the regression standardized predicted value (*x*-axis) and the actual values (*y*-axis) of the individual *Z*-score. The straight line represents the linear regression line, while the shadow on either side indicates the confidence intervals, illustrating the uncertainty around the predicted values. Each dot corresponds to an individual observation, reflecting the alignment between predicted and actual outcomes. The confidence intervals provide insight into the precision of the model’s predictions across the observed range. (**A–D**) The association between the reduced chance of verbal memory impairment (*Z*-scores < −1 SD) and volumes of the left dentate gyrus (DG), CA4 and CA3 is described by receiver operating characteristics curves (AUC = 0.712, 0.704 and 0.684, respectively); *N* = 84 (**E**).

Concerning hippocampal subfields, unique predictors of verbal learning *Z*-scores were the volumes of the left DG (*R*^2^ = 0.43; *b* = 5.2; SE = 1.7; *P* = 0.003), CA4 (*R*^2^ = 0.43; *b* = 5.8; SE = 2; *P* = 0.005) and CA3 (*R*^2^ = 0.41; *b* = 4.5; SE = 2; *P* = 0.03) (Fig. 4). In contrast, a greater volume of the right hippocampal tail was associated with worse verbal learning (*R*^2^ = 0.42; *b* = −2.6; SE = 1; *P* = 0.01). Using a backward stepwise method, the left DG and CA3 were included in the multiple regression model with the highest predictive power [*R*^2^ = 0.44; significant predictors: left DG (*b* = 10; SE = 4; *P* = 0.017)]. The multivariate backward stepwise method identified the left DG as the most predictive hippocampal subfield on verbal learning *Z-*scores (multivariate model *R*^2^ = 0.42; *b* = 0.47; SE = 0.2; *P* = 0.03).

To investigate the diagnostic value of hippocampal subfield volumes in patients with HS, a ROC curve analysis was conducted. The ROC curve showed that a greater volume of left DG, CA4 and CA3 was associated with a reduced chance of verbal memory impairment (verbal learning *Z*-score < −1 SD), with a marginally stronger association for the left DG than CA4, and CA3 (AUC = 0.712, 0.704 and 0.684, respectively; Fig. 4).

## Discussion

Using a large sample of TLE patients with HS and healthy subjects, we determined that verbal learning relates to the volume of distinct cortical regions and hippocampal subfields. Our results align with the role that these cortical areas and hippocampal structures have in human computational theories for episodic memory, and animal behavioural models. Further, the results support the thesis of a corticohippocampal network underpinning verbal memory encoding in individuals with TLE.

### Cortical regions involved in verbal learning

Grey matter volumes of the medial and dorsolateral PFC, STG and MTG, anterior and posterior cingulate of both hemispheres, and the left vlPFC and PTOj were associated with verbal learning performances (Figs 2 and 3); the greater the grey matter volumes, the better was verbal learning. Notably, the VBM analysis revealed consistent findings across both the entire cohort and the subgroup analysis focusing exclusively on patients with HS. As expected, the size of the significant clusters was reduced when the analysis was restricted to patients. This reduction may reflect decreased variability in grey matter volumes and verbal learning performances after excluding the HC group, underscoring the robustness of these findings within the patient population.

Historically, memory formation has been thought of as the direct transfer to the cortex of elements initially encoded by the hippocampus.^46^ There is now evidence that new memories are not simply transferred to the cortex. They are assimilated into cortical networks, called ‘schemas’, with memory encoding relying on the development and updating of these schemas. When a new experience occurs, this usually conflicts with pre-existing associations. The inclusion of new information into pre-existing cortical schemas represents the key mechanism for memory formation and learning.^47^ Prefrontal control of hippocampal encoding was suggested to have a critical role in learning, resolving the conflict between new information and pre-existing schemas.^46-48^ It seems likely that this accounts for the prefrontal cortices’ grey matter volumes associated with verbal learning in our findings.

The prefrontal cortex underwent great development in primates and humans,^49^ meeting the need for new areas to subserve further processing of sensorial information.^7^ Several prefrontal mechanisms contribute to schema integration and formation during learning. The first requirement for new information to be included in an existing schema is to hold the object of attention active and online among thousands of neural attractors in the cortex.^7^ Through a top-down neural control mechanism, the PFC enhances the selection of populations of neurons useful for the various steps of memory encoding, favouring an efficient allocation of neural resources during learning.^50^ Further, the number of active memories that are integrated into a schema is affected by neural processes such as synaptic facilitation of the type found in the PFC.^51^ This is ascribed to the recurrent collateral connections to nearby neurons which is a typical feature of PFC architecture.^51^ Organization and strategies are other important mechanisms for dealing with large amounts of verbal information^52^; again, these processes were found to be related to PFC.^15^

The pivotal role of PFC during verbal learning is further supported by animal and human studies. During memory encoding, a striking learning-associated upregulation of genes associated with plasticity was demonstrated in the mPFC of rats,^48^ with pharmacological interventions targeting this area preventing learning.^48^ In a similar way, human investigations demonstrated a maximal increased metabolic activity in the mPFC and dlPFC during the execution of auditory-verbal memory tests similar to those used in this study,^15^ with lesions located in the dlPFC and mPFC being specifically related to verbal learning and encoding deficits in patients with strokes, tumours and haemorrhages.^16^ Further, our results align with findings derived from intracranial electrophysiological recording.^6^ During verbal memory encoding, increased low-band neuronal activity was found in cortical regions of the left vlPFC that was consistent with the hotspot of the left vlPFC grey matter volume related to verbal learning in our study.^6^

The posterior STG and MTG represented the main foci of grey matter volumes of the posterior cortex that were associated with verbal learning in our study. These areas, which overlap the Brodmann areas 41, 42, 21 and 22, represent primary auditory receptive and auditory association cortical areas.^15^ Their role in verbal encoding is attributed to the relationship between the acoustic and phonemic properties of words and verbal learning, with acoustic similarities being an acknowledged factor associated with the learning of words.^8-10^ Moreover, these regions may contribute to the learning of verbal material through their function as a storage component of verbal working memory and their active role in rehearsal, a recognized mechanism used during immediate verbal recall.^12,53^ In both functional and lesion studies, researchers demonstrated that these cortical regions may play a major role in verbal memory performances.^6,15,54^ Increased cerebral metabolism,^15^ and recorded neural activity,^6^ were previously described in these cortical regions during the execution of verbal learning tasks, and lesions involving these cortical hubs related to deficits in verbal learning and encoding.^54^ Interestingly, the left PTOj was also related to verbal learning in our study. This region of the posterior cortex has been suggested to be a short-term memory system for human auditory-verbal memory;^7^ that is, patients with damage to this system cannot repeat a heard string of words.^7^

The anterior and posterior cingulate (retrosplenial) cortex was associated with verbal learning in our study. A possible explanation may lie in the connections that ACC and PCC have with the parahippocampal gyrus (representing a direct route to the hippocampus via the entorhinal cortex), the auditory superior temporal gyrus, the dorsal bank of the superior temporal sulcus and with areas of the mPFC.^28,29^ Interestingly, these regions are also those identified as significant by our morphometric analysis. Therefore, we suggest that the cingulate cortices are of pivotal importance to connect the two systems—cortical and hippocampal—and to allow them to efficiently work together, as illustrated by evidence that compensatory network shifts involving the cingulate in response to hippocampal dysfunction.^55-57^ This hypothesis is also corroborated by previous studies showing that GABA cingulate interneuron activity was highly involved in synchronizing network activity and establishing functional oscillations that are associated with information processing and learning.^58^ Lesions in both the ACC and PCC were related to deficits of verbal learning,^16,59^ with functional studies further supporting the role of ACC and PCC in encoding and learning of verbal material.^15,60^

### Hippocampal subfields involved in verbal learning

We determined that verbal learning is related to the volumes of the left DG, CA4 and CA3 hippocampal subfields. Patients with reduced volume of the left DG, CA4 and CA3 had worse verbal learning, with the left DG being the main driver.

The DG has a key role in pattern separation and, consequently, in learning and memory encoding. Briefly, signals from the entorhinal cortex are decorrelated and augmented in the DG, before the inputs are presented to the CA3 associative network.^7,35,39,61,62^ Larger volumes of DG were related to a better lure discrimination index in humans.^63^ Our results identified the DG volume as having the strongest association with verbal learning performance, suggesting its role as the main encoding hippocampal unit in verbal memory. Previous pathological investigations confirmed our theory. Degeneration of DG has been described as the strongest predictor of worse verbal learning performances.^21^ The DG’s encoding role relies on neurogenesis,^22,23,64^ with the suggestion that pattern separation can be facilitated by new granule cells’ formation.^22^ Since neurogenesis is highly influenced by granule-cell density in DG,^23^ further impairment of the learning processes in verbal memory can be explained by the reduced neurogenesis in patients with more atrophic, less granule-cell-populated, DGs.

The anatomical and functional connection between DG and CA3 is another fundamental aspect of learning.^7^ Particularly, the mossy fibre system is necessary for the optimal storage of new information in the CA3 associative network.^7,36^ Thus, a significant relationship between the extent of CA3 atrophy and verbal learning performances was anticipated. The importance of CA3 for verbal learning can reside in the associative role that this subfield exerts on inputs from the DG (via the mossy fibre system) and entorhinal cortex (via the perforant pathway), with CA3 demonstrating pattern separation activity under some circumstances and pattern completion activity under others.^39^ Consistent with this theory, pathological studies showed a significant association between severe verbal learning deficits and the lack of a preserved mossy fibre pathway.^21^ Although the CA3 associative role has been suggested to be more important for recall,^35^ pattern completion (and pattern separation under some circumstances) is still a fundamental aspect of learning. The results of this study are concordant with this hypothesis and the suggestion that CA3 is fundamental in some learning tests.^35^

Reduced volumes of the left CA4 predicted worse verbal learning scores. CA4 contributes to pattern separation and context recognition during learning.^37,38^ Its cells are thought to play a complementary role in pattern separation by preprocessing the inputs that will be transmitted to CA3.^37^ Thus, CA4 can contribute to verbal learning by preprocessing information from the DG and directed to CA3, with reduced CA4 volumes being related to worse verbal learning performances. Our results align with previous pathological data. Worse verbal learning performance was seen in patients with degeneration of CA4 than in patients who showed degeneration of CA1.^20^

Although there is evidence that the left and right hippocampi in humans might work together to process qualitatively different information relevant to visual memory tasks,^65^ it is still debated whether task lateralization exists for verbal memory. Our results support a left hippocampal lateralization for verbal memory with verbal learning impairment being related to reduced volumes of the left DG, CA4 and CA3 subfields.^17,66^ Further, we observed an inverse relationship between verbal learning and right hippocampal volumes, with the increased right hippocampal tail volume being related to worse verbal learning *Z*-scores. In a recent longitudinal study, preserved preoperative network and function before surgery facilitated memory adequacy postsurgery, with memory adequacy relying on the ipsilateral connectivity of structures situated close to the resected areas.^57^ While for other types of memory, such as associative memory,^67^ a compensatory role of the nonaffected contralateral hippocampus was hypothesized, our results concur with the previously proposed functional adequacy model for verbal memory,^34,57^ postulating that the contralateral nonaffected (nondominant) hippocampus is not able to compensate for the affected left-sided (dominant) HS-related verbal memory deficit. These observations are in keeping with the postoperative functional outcomes reported after left ATLR with verbal memory decline in up to 60% of patients,^3,4,34,68^ and a substantial overlap (20% of patients)^69^ between the pre- and postoperative verbal memory scores in patients receiving right ATLR.^70^

### Cortical–hippocampal networks underpin human verbal encoding

Our results suggest a cortical–hippocampal network for verbal memory in individuals with TLE. The theory states that prefrontal cortices receive auditory-verbal information from primary and associative auditory cortices of STG and MTG. Via the cingulum, prefrontal cortices and left hippocampus cooperate to assimilate the new information into pre-existing schemas. Within the left hippocampus, the DG and CA4 are fundamental to decorrelate and augment the information through pattern separation, favouring their storage in the CA3 associative network. A schematic representation of our theory is available in the graphical abstract.

The proposed network aligns with the architecture of the involved cortical hubs and the connectivity of white matter tracts. Primary sensory cortical areas have a well-developed granule-cell layer which relies on their role of input first-processing units where representations of the inputs are sparser.^7^ The architecture of the PFC differs from that of sensory cortices in the complexity of the dendritic structure which endows its neurons with higher computational capability, making their recurrent collateral connections crucial to increasing memory capacity and encoding.^7,71^ The proisocortical structure of the cingulum favours forming connectional bridges between the neocortical areas of both hemispheres and the allocortex of the left hippocampus.^72^ Higher-order auditory areas encircle and receive verbal information from the primary auditory cortex. At least two projections emerge from the higher-order auditory areas: one projection is thought to be important for sound localization and it targets the dlPFC; the second projection is involved in processing complex sounds, including linguistic function in humans, and terminates in vlPFC and mPFC.^73^ The arcuate fasciculus has a key role, interconnecting the superior temporal cortex to PTOj and prefrontal cortices.^73^ The cingulum is a collection of axons coursing deep within the cingulate cortices (ACC and PCC) and parahippocampal gyrus. It is the principal pathway carrying information from higher-order sensorial areas in the temporal lobe, prefrontal cortices and PTOj to the hippocampus.^73^ The fornix, via telencephalic and diencephalic structures, connect the cingulate cortices and mPFC to the hippocampus.^73^ This compact fibre bundle is essential for the bidirectional control and effective interplay between mPFC, cingulate cortices and hippocampus which is fundamental for schemas’ integration and update. Finally, to integrate the information of the two halves of the cerebral cortex, tracts that interconnect the two sides of the brain course through the corpus callosum.^73^

### Limitations and future prospects

Our study has several strengths, including the use of one of the largest series of HCs and individuals with TLE and HS, the employment of normalized neuropsychological data derived from standardized neuropsychological testing, high-resolution MRI data acquired on the same scanner, robust methodology (Bonferroni corrected analyses) and the correction of our findings for the main predictable confounders (age, sex, handedness, history and duration of epilepsy, frequency of seizures, presence and side of HS and number of ASM). The human corticohippocampal verbal memory network here proposed is supported by animal models, human lesion and functional studies and anatomical and brain connectivity evidence. However, several limitations must be discussed. With the aim of discovering true associations between verbal learning and grey matter volumes of the cerebral cortex and hippocampal subfields, we restricted our analyses to HCs and individuals with TLE who underwent standardized neuropsychological evaluation and MRI data acquisition on the same scanner. This reduced any potential biases caused by different MRI acquisition protocols and variations related to diverse neuropsychological testing. Validation in future studies is encouraged. Second, given the clinical relevance of verbal learning impairments in patients with HS,^5^ we focused our hypothesis specifically on this cognitive domain. While this targeted approach allowed us to concentrate on a key area of cognitive dysfunction, it limited our ability to assess relationships with other cognitive functions, such as fluency, linguistic phonemic and semantic processing or executive control. Future studies should expand upon these findings to clarify the role of the identified cortical and hippocampal structures in supporting verbal memory within a broader cognitive framework that integrates language, memory processes and executive control. Further, even if widely used and validated, the subfield segmentation method we employed on 3T MRI data is still the best approximation available to investigate hippocampal subfields. Future studies may benefit from using 7T MRI images which will allow better discrimination between the different hippocampal subfields and quantification of their volumes.

### Clinical implications

The current study demonstrated that verbal learning, in patients with TLE, is strongly related to the volume of distinct regions of the prefrontal, temporal and cingulate cortices and to the left DG, CA4 and CA3 hippocampal subfields. This finding is important. Firstly, a cortical–hippocampal network for verbal memory may be hypothesized for these patients, providing the basis for a better understanding of human verbal memory, with biomarkers that are important for both clinical and research perspectives. Secondly, this study may provide attractive targets for forthcoming modulating therapies. Up to now, direct stimulation of the lateral temporal cortex has been shown to successfully improve episodic memory encoding.^74^ Future studies targeting the prefrontal cortices and/or left PTOj may lead to interesting results. Thirdly, we and others previously showed that verbal memory MRI activation in the posterior hippocampus is protective against verbal memory decline after language-dominant ATLR, and that sparing the posterior 45% of the speech-dominant hippocampus mitigated the risk of verbal memory decline.^18,66,75^ Future work will analyse the impact of sparing or resecting posterior left DG, CA4 and CA3 on verbal memory.

## Data Availability

Anonymized raw data that support the findings of this study are available from the corresponding author upon reasonable request.

## References

[1] Bakker A, Kirwan CB, Miller M, Stark CEL. Pattern separation in the human hippocampal CA3 and dentate gyrus. Science. 2008;319(5870):1640–1642. 18356518 10.1126/science.1152882 PMC2829853

[2] De Tisi J, Bell GS, Peacock JL, et al The long-term outcome of adult epilepsy surgery, patterns of seizure remission, and relapse: A cohort study. Lancet. 2011;378(9800):1388–1395. 22000136 10.1016/S0140-6736(11)60890-8

[3] Bauman K, Devinsky O, Liu AA. Temporal lobe surgery and memory: Lessons, risks, and opportunities. Epilepsy Behav. 2019;101: 106596. 31711868 10.1016/j.yebeh.2019.106596 PMC6885125

[4] Rausch R, Kraemer S, Pietras CJ, Le M, Vickrey BG, Passaro EA. Early and late cognitive changes following temporal lobe surgery for epilepsy. Neurology. 2003;60(6):951–959. 12654959 10.1212/01.wnl.0000048203.23766.a1

[5] Baxendale S, Thompson P, Harkness W, Duncan J. Predicting memory decline following epilepsy surgery: A multivariate approach. Epilepsia. 2006;47(11):1887–1894. 17116029 10.1111/j.1528-1167.2006.00810.x

[6] Topçu Ç, Marks VS, Saboo KV, et al Hotspot of human verbal memory encoding in the left anterior prefrontal cortex. EBioMedicine. 2022;82:104135. 35785617 10.1016/j.ebiom.2022.104135 PMC9254338

[7] Rolls ET . Cerebral cortex. Principles of Operation; 2016.

[8] Baddeley AD . Short-term memory for word sequences as a function of acoustic, semantic and formal similarity. Q J Exp Psychol. 1966;18(4):362–365. 5956080 10.1080/14640746608400055

[9] Shallice T, Papagno C. Impairments of auditory-verbal short-term memory: Do selective deficits of the input phonological buffer exist? Cortex. 2019;112:107–121. 30414628 10.1016/j.cortex.2018.10.004

[10] Zatorre RJ, Samson S. Role of the right temporal neocortex in retention of pitch in auditory short-term memory. Brain. 1991;114(6):2403–2417. 1782523 10.1093/brain/114.6.2403

[11] Koelsch S, Schulze K, Sammler D, Fritz T, Müller K, Gruber O. Functional architecture of verbal and tonal working memory: An FMRI study. Hum Brain Mapp. 2009;30(3):859–873. 18330870 10.1002/hbm.20550 PMC6871123

[12] Hickok G, Buchsbaum B, Humphries C, Muftuler T. Auditory–motor interaction revealed by fMRI: Speech, music, and working memory in area spt. J Cogn Neurosci. 2003;15(5):673–682. 12965041 10.1162/089892903322307393

[13] Davidson PSR, Troyer AK, Moscovitch M. Frontal lobe contributions to recognition and recall: Linking basic research with clinical evaluation and remediation. J Int Neuropsychol Soc. 2006;12(2):210–223. 16573855 10.1017/S1355617706060334

[14] Bor D, Cumming N, Scott CEL, Owen AM. Prefrontal cortical involvement in verbal encoding strategies. Eur J Neurosci. 2004;19(12):3365–3370. 15217392 10.1111/j.1460-9568.2004.03438.x

[15] Grasby PM, Frith CD, Friston KJ, Bench C, Frackowiak RSJ, Dolan RJ. Functional mapping of brain areas implicated in auditory—verbal memory function. Brain. 1993;116(1):1–20. doi: 10.1093/brain/116.1.1 8453452

[16] Alexander MP, Stuss DT, Fansabedian N. California verbal learning test: Performance by patients with focal frontal and non-frontal lesions. Brain. 2003;126(6):1493–1503. 12764068 10.1093/brain/awg128

[17] Richardson MP, Strange BA, Thompson PJ, Baxendale SA, Duncan JS, Dolan RJ. Pre-operative verbal memory fMRI predicts post-operative memory decline after left temporal lobe resection. Brain. 2004;127(11):2419–2426. 15459025 10.1093/brain/awh293

[18] Sidhu MK, Stretton J, Winston GP, et al Memory network plasticity after temporal lobe resection: A longitudinal functional imaging study. Brain. 2016;139(2):415–430. 26754787 10.1093/brain/awv365 PMC4805088

[19] Krings T, Topper R, Willmes K, Reinges MHT, Gilsbach JM, Thron A. Activation in primary and secondary motor areas in patients with CNS neoplasms and weakness. Neurology. 2002;58(3):381–390. 11839836 10.1212/wnl.58.3.381

[20] Rodrigues GR, Kandratavicius L, Peixoto-Santos JE, et al Increased frequency of hippocampal sclerosis ILAE type 2 in patients with mesial temporal lobe epilepsy with normal episodic memory. Brain. 2015;138(6):e359. 25416180 10.1093/brain/awu340 PMC4614134

[21] Prada Jardim A, Liu J, Baber J, et al Characterising subtypes of hippocampal sclerosis and reorganization: Correlation with pre and postoperative memory deficit. Brain Pathol. 2018;28(2):143–154. 28380661 10.1111/bpa.12514 PMC5893935

[22] Nakashiba T, Cushman JD, Pelkey KA, et al Young dentate granule cells mediate pattern separation, whereas old granule cells facilitate pattern completion. Cell. 2012;149(1):188–201. 22365813 10.1016/j.cell.2012.01.046 PMC3319279

[23] Coras R, Siebzehnrubl FA, Pauli E, et al Low proliferation and differentiation capacities of adult hippocampal stem cells correlate with memory dysfunction in humans. Brain. 2010;133(11):3359–3372. 20719879 10.1093/brain/awq215

[24] Blümcke I, Thom M, Aronica E, et al International consensus classification of hippocampal sclerosis in temporal lobe epilepsy: A task force report from the ILAE commission on diagnostic methods. Epilepsia. 2013;54(7):1315–1329. 23692496 10.1111/epi.12220

[25] Thom M, Liagkouras I, Elliot KJ, et al Reliability of patterns of hippocampal sclerosis as predictors of postsurgical outcome. Epilepsia. 2010;51(9):1801–1808. 20738385 10.1111/j.1528-1167.2010.02681.x

[26] Bigras C, Shear PK, Vannest J, Allendorfer JB, Szaflarski JP. The effects of temporal lobe epilepsy on scene encoding. Epilepsy Behav. 2013;26(1):11–21. 23207513 10.1016/j.yebeh.2012.10.017 PMC3529961

[27] Barnett AJ, Park MTM, Pipitone J, Chakravarty MM, McAndrews MP. Functional and structural correlates of memory in patients with mesial temporal lobe epilepsy. Front Neurol. 2015;6:103. 26029159 10.3389/fneur.2015.00103 PMC4429573

[28] Kobayashi M, Kanai T. Vertebral body infarction revealed by diffusion-weighted magnetic resonance imaging. J Neurol. 2013;260(4):1160–1162. 23355178 10.1007/s00415-013-6843-0

[29] Kobayashi Y, Amaral DG. Macaque monkey retrosplenial cortex: III. Cortical efferents. J Comp Neurol. 2007;502(5):810–833. 17436282 10.1002/cne.21346

[30] Ashburner J, Friston KJ. Voxel-based morphometry—The methods. Neuroimage. 2000;11(6):805–821. 10860804 10.1006/nimg.2000.0582

[31] Sämann PG, Iglesias JE, Gutman B, et al FreeSurfer-based segmentation of hippocampal subfields: A review of methods and applications, with a novel quality control procedure for ENIGMA studies and other collaborative efforts. Hum Brain Mapp. 2022;43(1):207–233. 33368865 10.1002/hbm.25326 PMC8805696

[32] Iglesias JE, Augustinack JC, Nguyen K, et al A computational atlas of the hippocampal formation using ex vivo, ultra-high resolution MRI: Application to adaptive segmentation of in vivo MRI. Neuroimage. 2015;115:117–137. 25936807 10.1016/j.neuroimage.2015.04.042 PMC4461537

[33] Winston GP, Cardoso MJ, Williams EJ, et al Automated hippocampal segmentation in patients with epilepsy: Available free online. Epilepsia. 2013;54(12):2166–2173. 24151901 10.1111/epi.12408 PMC3995014

[34] Stroup E, Langfitt J, Berg M, McDermott M, Pilcher W, Como P. Predicting verbal memory decline following anterior temporal lobectomy (ATL). Neurology. 2003;60(8):1266–1273. 12707428 10.1212/01.wnl.0000058765.33878.0d

[35] Rolls ET . The storage and recall of memories in the hippocampo-cortical system. Cell Tissue Res. 2018;373(3):577–604. 29218403 10.1007/s00441-017-2744-3 PMC6132650

[36] Treves A, Rolls ET. Computational constraints suggest the need for two distinct input systems to the hippocampal CA3 network. Hippocampus. 1992;2(2):189–199. 1308182 10.1002/hipo.450020209

[37] Hunsaker MR, Kesner RP. The operation of pattern separation and pattern completion processes associated with different attributes or domains of memory. Neurosci Biobehav Rev. 2013;37(1):36–58. 23043857 10.1016/j.neubiorev.2012.09.014

[38] Carey D, Nolan H, Kenny RA, Meaney J. Dissociable age and memory relationships with hippocampal subfield volumes in vivo: Data from the Irish Longitudinal Study on Ageing (TILDA). Sci Rep. 2019;9(1):10981. 31358771 10.1038/s41598-019-46481-5 PMC6662668

[39] Yassa MA, Stark CEL. Pattern separation in the hippocampus. Trends Neurosci. 2011;34(10):515–525. 21788086 10.1016/j.tins.2011.06.006 PMC3183227

[40] Baxendale SA, Thompson PJ. The clinical utility of a memory specialization index in epilepsy surgery patients with unilateral hippocampal sclerosis. Epilepsia. 2021;62(7):1584–1593. 33971016 10.1111/epi.16919

[41] von Elm E, Altman DG, Egger M, Pocock SJ, Gøtzsche PC, Vandenbroucke JP. Strengthening the reporting of observational studies in epidemiology (STROBE) statement: Guidelines for reporting observational studies. BMJ. 2007;335(7624):806–808. 17947786 10.1136/bmj.39335.541782.AD PMC2034723

[42] Petersen SE, Seitzman BA, Nelson SM, Wig GS, Gordon EM. Principles of cortical areas and their implications for neuroimaging. Neuron. 2024;112(17):2837–2853. 38834069 10.1016/j.neuron.2024.05.008 PMC13339775

[43] Postma TS, Cury C, Baxendale S, et al Hippocampal shape is associated with memory deficits in temporal lobe epilepsy. Ann Neurol. 2020;88(1):170–182. 32379905 10.1002/ana.25762 PMC8432153

[44] Cook MJ, Fish DR, Shorvon SD, Straughan K, Stevens JM. Hippocampal volumetric and morphometric studies in frontal and temporal lobe epilepsy. Brain. 1992;115(4):1001–1015. 1393499 10.1093/brain/115.4.1001

[45] Cardoso MJ, Modat M, Wolz R, et al Geodesic information flows: Spatially-variant graphs and their application to segmentation and fusion. IEEE Trans Med Imaging. 2015;34(9):1976–1988. 25879909 10.1109/TMI.2015.2418298

[46] Preston AR, Eichenbaum H. Interplay of hippocampus and prefrontal cortex in memory. Curr Biol. 2013;23(17):R764–R773. 24028960 10.1016/j.cub.2013.05.041 PMC3789138

[47] DeVito LM, Lykken C, Kanter BR, Eichenbaum H. Prefrontal cortex: Role in acquisition of overlapping associations and transitive inference. Learn Mem. 2010;17(3):161–167. 20189961 10.1101/lm.1685710 PMC2832922

[48] Tse D, Takeuchi T, Kakeyama M, et al Schema-dependent gene activation and memory encoding in neocortex. Science. 2011;333(6044):891–895. 21737703 10.1126/science.1205274

[49] Pandya D, Seltzer B, Petrides M, Cipolloni PB. Cerebral cortex. Oxford University Press; 2015.

[50] Rolls ET, Deco G. Computational neuroscience of vision. Oxford University Press; 2002.

[51] Rolls ET, Dempere-Marco L, Deco G. Holding multiple items in short term memory: A neural mechanism. PLoS One. 2013;8(4):e61078. 23613789 10.1371/journal.pone.0061078 PMC3628858

[52] Shimamura AP, Janowsky JS, Squire LR. What is the role of frontal lobe damage in memory disorders? Frontal lobe function and dysfunction. Oxford University Press; 1991:173–195.

[53] Becker JT, MacAndrew DK, Fiez JA. A comment on the functional localization of the phonological storage subsystem of working memory. Brain Cogn. 1999;41(1):27–38. 10536084 10.1006/brcg.1999.1094

[54] Shallice T, Vallar G. The impairment of auditory-verbal short-term storage. Neuropsychological impairments of short-term memory. Cambridge University Press; 1990:11–53.

[55] McCormick C, Protzner AB, Barnett AJ, Cohn M, Valiante TA, McAndrews MP. Linking DMN connectivity to episodic memory capacity: What can we learn from patients with medial temporal lobe damage? Neuroimage Clin. 2014;5:188–196. 25068108 10.1016/j.nicl.2014.05.008 PMC4110351

[56] Barnett AJ, Man V, McAndrews MP. Parcellation of the hippocampus using resting functional connectivity in temporal lobe epilepsy. Front Neurol. 2019;10:920. 31507522 10.3389/fneur.2019.00920 PMC6714062

[57] Fleury MN, Binding LP, Taylor P, et al Predictors of long-term memory and network connectivity 10 years after anterior temporal lobe resection. Epilepsia. 2024;65(9):2641–2661. 38990127 10.1111/epi.18058 PMC7619481

[58] Durazzo TC, Meyerhoff DJ. GABA concentrations in the anterior cingulate and dorsolateral prefrontal cortices: Associations with chronic cigarette smoking, neurocognition, and decision making. Addict Biol. 2021;26(3):e12948. 33860602 10.1111/adb.12948 PMC8697713

[59] Chang YL, Jacobson MW, Fennema-Notestine C, et al Level of executive function influences verbal memory in amnestic mild cognitive impairment and predicts prefrontal and posterior cingulate thickness. Cereb Cortex. 2010;20(6):1305–1313. 19776343 10.1093/cercor/bhp192 PMC2912652

[60] Lim TS, Hong YH, Choi JY, Kim HS, Moon SY. Functional investigation of bilateral posterior cingulate gyri using multivoxel MR spectroscopy. Eur Neurol. 2012;67(5):279–286. 22472573 10.1159/000336834

[61] Leutgeb JK, Leutgeb S, Moser MB, Moser EI. Pattern separation in the dentate gyrus and CA3 of the hippocampus. Science. 2007;315(5814):961–966. 17303747 10.1126/science.1135801

[62] Rolls ET . The hippocampus, ventromedial prefrontal cortex, and episodic and semantic memory. Prog Neurobiol. 2022;217:102334. 35870682 10.1016/j.pneurobio.2022.102334

[63] Riphagen JM, Schmiedek L, Gronenschild EHBM, et al Associations between pattern separation and hippocampal subfield structure and function vary along the lifespan: A 7 T imaging study. Sci Rep. 2020;10(1):7572. 32371923 10.1038/s41598-020-64595-z PMC7200747

[64] Clelland CD, Choi M, Romberg C, et al A functional role for adult hippocampal neurogenesis in spatial pattern separation. Science. 2009;325(5937):210–213. 19590004 10.1126/science.1173215 PMC2997634

[65] Lee CH, Ryu J, Lee SH, Kim H, Lee I. Functional cross-hemispheric shift between object-place paired associate memory and spatial memory in the human hippocampus. Hippocampus. 2016;26(8):1061–1077. 27009679 10.1002/hipo.22587 PMC5074286

[66] Bonelli SB, Powell RHW, Yogarajah M, et al Imaging memory in temporal lobe epilepsy: Predicting the effects of temporal lobe resection. Brain. 2010;133(4):1186–1199. 20157009 10.1093/brain/awq006 PMC2850579

[67] Finke C, Bruehl H, Düzel E, Heekeren HR, Ploner CJ. Neural correlates of short-term memory reorganization in humans with hippocampal damage. J Neurosci. 2013;33(27):11061–11069. 23825411 10.1523/JNEUROSCI.0744-13.2013 PMC6618604

[68] Ivnik RJ, Sharbrough FW, Laws ER. Anterior temporal lobectomy for the control of partial complex seizures: Information for counseling patients. Mayo Clin Proc. 1988;63(8):783–793. 3398596 10.1016/s0025-6196(12)62358-1

[69] Sherman EMS, Wiebe S, Fay-Mcclymont TB, et al Neuropsychological outcomes after epilepsy surgery: Systematic review and pooled estimates. Epilepsia. 2011;52(5):857–869. 21426331 10.1111/j.1528-1167.2011.03022.x

[70] Alpherts WCJ, Vermeulen J, van Rijen PC, da Silva FHL, van Veelen CWM. Verbal memory decline after temporal epilepsy surgery?: A 6-year multiple assessments follow-up study. Neurology. 2006;67(4):626–631. 16924016 10.1212/01.wnl.0000230139.45304.eb

[71] Elston GN, Benavides-Piccione R, Elston A, et al Specializations of the granular prefrontal cortex of primates: Implications for cognitive processing. Anat Rec A Discov Mol Cell Evol Biol. 2006;288A(1):26–35. 10.1002/ar.a.20278 16342214

[72] Rolls ET . The cingulate cortex and limbic systems for emotion, action, and memory. Brain Struct Funct. 2019;224(9):3001–3018. 31451898 10.1007/s00429-019-01945-2 PMC6875144

[73] Martin J . Neuroanatomy text and atlas. 5th edn. McGraw Hill; 2020.

[74] Ezzyat Y, Wanda PA, Levy DF, et al Closed-loop stimulation of temporal cortex rescues functional networks and improves memory. Nat Commun. 2018;9(1):365. 29410414 10.1038/s41467-017-02753-0 PMC5802791

[75] Sone D, Ahmad M, Thompson PJ, et al Optimal surgical extent for memory and seizure outcome in temporal lobe epilepsy. Ann Neurol. 2022;91(1):131–144. 34741484 10.1002/ana.26266 PMC8916104

